# Iridium nanoparticles supported on hierarchical porous N-doped carbon: an efficient water-tolerant catalyst for bio-alcohol condensation in water

**DOI:** 10.1038/srep21365

**Published:** 2016-02-25

**Authors:** Di Liu, Xiufang Chen, Guoqiang Xu, Jing Guan, Quan Cao, Bo Dong, Yunfei Qi, Chunhu Li, Xindong Mu

**Affiliations:** 1 Key Laboratory of Bio-based Materials, Qingdao Institute of Bioenergy and Bioprocess Technology, Chinese Academy of Sciences, Qingdao 266101, China; 2 Key Laboratory of Marine Chemistry Theory and Technology of Ministry of Education, Ocean University of China, Qingdao 266100, China

## Abstract

Nitrogen-doped hierarchical porous carbons were synthesized successfully by a controllable one-pot method using glucose and dicyandiamide as carbon source and nitrogen source via hydrothermal carbonization process. The nitrogen-doped materials, possessing high nitrogen content (up to 7 wt%), large surface area (>320 m^2^ g^−1^) and excellent hierarchical nanostructure, were employed as catalyst supports for immobilization of iridium nanoparticles for bio-alcohol condensation in water. The introduction of nitrogen atoms into the carbon framework significantly improved iridium nanoparticles dispersion and stabilization. The novel iridium catalysts exhibited superior catalytic activity in the aqueous phase condensation of butanol, offering high butanol conversion of 45% with impressive 2-ethylhexanol selectivity of 97%. The heterogeneous catalysts had great advantages of easy recovery and high catalytic stability. The outstanding catalytic performance could be attributed to excellent dispersion of iridium nanoparticles, stronger iridium-support interactions and interaction of nitrogen species with alcohol substrates.

Recently, condensation of bio-alcohols has attracted great research interest from both industrial and academic chemists, which has promising application in producing high-value chemicals from regenerative resources^1^,^2^,^3^,^4^,^5^. Alcohol condensation, also known as the Guerbet reaction, is a crucial industrial process to increase the value of short chain alcohols^6^. In the past, the reaction was always performed in gas phase or organic solvents^7^,^8^. From the economic and environmental standpoints, water undoubtedly has more advantages than other solvents. Particularly, crude bio-alcohols were usually produced by fermentation in aqueous solutions, which was quite difficult and expensive to separate the bio-alcohols from water by purification^8^. Thus, it is desirable to transform bio-alcohols to high-value chemicals directly in the fermentation solution. Moreover, water is the sole by-product in the alcohol condensation process in theory. However, the formation of water during condensation process is often harmful to many catalysts in the reaction system^7^. So the development of an active water-tolerant catalyst for alcohol condensation in aqueous phase remains a key challenge.

During the past decades, some homogeneous catalysts have been investigated for the alcohol condensation^9^,^10^,^11^,^12^,^13^. For example, iridium catalysts, such as [Ir(acac)-(cod)] and [Cp*IrCl_2_]_2_, were found to catalyze the condensation of primary alcohols to higher-alcohols with alcohol or p-xylene as solvent^10^. Alkene additives were needed to add to the reaction system for stabilizing the Ir intermediates by the formation of weakly coordinated ligands. RhCl_3_.3H_2_O/4P(C_4_H_9_)_3_ was also examined as an active catalyst for alcohol condensation by generation of stable hydride metal complexes^11^. In contrast to homogeneous catalysts, heterogeneous catalysts could be recovered conveniently by simple filtration, which are more suitable for large-scale application. Thus, much effort has also been focused on exploring heterogeneous catalysts for alcohol condensation^14^,^15^,^16^,^17^. For instance, Marcu *et al.* used Mg-Al mixed oxide supported metals (such as Pd, Ag, Mn, Fe, Cu, Sm, Yb) as the catalysts for the one-pot conversion of ethanol to butanol^15^. However, the temperature of the reaction was over 200 ^o^C, and the catalysts would be deactivated by water formed during the reaction. Carlini *et al.* reported that Pd/C efficiently catalyzed condensation of butanol to 2-ethylhexanol in good yields in the presence of BuONa^14^. Unfortunately, it would become inactive with the gradual hydrolysis of BuONa to NaOH. Other Ru and Rh based catalysts were also examined as active catalysts for alcohol condensation to reach high selectivity^17^. However, most reactions were usually performed in organic solvent in order to avoid the detrimental impacts of water. The efficient catalytic system for alcohol condensation in aqueous phase has been rarely explored. In our previous work, we found that the combination of IrCl_3_ with N-containing ligands such as 1, 10-phenanthroline could promote the butanol condensation in aqueous phase^18^. Simple immobilization of the catalysts over activated carbon showed very poor activity for the self-condensation of butanol in aqueous phase. But when such Ir/C catalyst was combined with 1, 10-phenanthroline, which was directly added to the reactant mixture, the transformation could be promoted greatly. These results indicated that N-containing ligands played a crucial role in the catalytic process. So, a new efficient, highly water-tolerant, and heterogeneous catalyst containing nitrogen ligands for alcohol condensation in water would be highly desirable.

Carbon nanomaterials have unique physical, chemical and electronic properties, such as high surface area, chemical inertness and good conductivity, which displayed a great potential in the carbon dioxide adsorption, catalysis and other fields^19^,^20^,^21^. However, pure carbon materials have poor functional groups and hydrophilicity, which limit their practical applications. Functionalization, such as nitrogen atom doping, would enhance intrinsic properties or add new features to carbon materials^22^,^23^. The nitrogen atom is adjacent to the carbon atom in the periodic table, so it can substitute the carbon atom in the materials. The doping of electron-rich nitrogen in the carbon architecture may greatly change the surface structure, increase the hydrophilicity, enhance π-binding ability and improve electron transport rate of materials, thus make the functional materials as fascinating supports for heterogeneous catalysis^24^,^25^,^26^. Recently, a series of methodologies have been developed to prepare nitrogen-doped carbons^27^,^28^,^29^,^30^,^31^. Among them, *in-situ* doping of carbons with nitrogen-rich precursors allows access to uniform dopant distribution. For example, Wang *et al.* prepared a porous nitrogen-doped carbon nanomaterial with glucose and nitrogen-containing ionic liquid as precursors by hydrothermal carbonization (HTC) method, which was an excellent support to construct Pd catalyst in the selective aerobic oxidation of alcohols and hydrocarbons^32^. However, from the point of view of the economy, ionic liquid isn’t an adaptive source of nitrogen ligands, which is too expensive and could not be synthesized using facile methods.

In this work, focusing on supported Ir nanoparticles as the catalytic agent, we synthesized a kind of nitrogen-doped carbon nanomaterials with glucose and dicyandiamide (DCDA) as carbon and nitrogen sources by HTC method and explored the as-made nanomaterials as basic supports to immobilize Ir catalyst for alcohol condensation in water. Commonly available inexpensive DCDA was an interesting nitrogen additive to fabricate nitrogen-doped carbon materials, which is water soluble and nitrogen rich. A range of nitrogen-doped carbon nanomaterials were produced by adjusting DCDA addition amounts and post-carbonization temperatures. The doped materials had large surface area with hierarchical porous network and high nitrogen content. We also showed the efficient self-condensation of butanol in water by these novel Ir-based catalysts, which gave high butanol conversion of 45% with impressive 2-ethylhexanol selectivity of 97%. The advantages including high activity, high stability, good recyclability and simple synthetic approach enabled the novel Ir-based nanomaterials as attractive heterogeneous catalysts for industrial application.

## Results and Discussion

### Characterizations of nanostructured nitrogen-doped carbon materials

**Figure 1:**
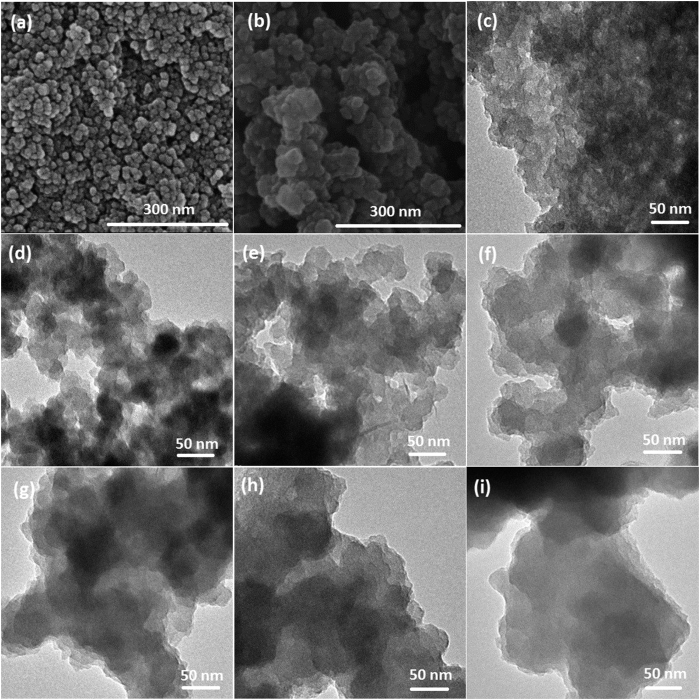
SEM images of (**a**) MC-550, (**b**) 0.4DMC-550. TEM images of (**c**) MC-550, (**d**) 0.25DMC-550, (**e**) 0.4DMC-550, (**f**) 0.5DMC-550, (**g**) 0.6DMC-550 (**h**) 0.7DMC-550, (**i**) 0.8DMC-550.

Nitrogen-doped carbons were synthesized by a facile HTC approach with DCDA as nitrogen source, which resulted in the formation of nanoporous structure and the uniform inclusion of nitrogen in the carbon matrix simultaneously. The nitrogen-doped carbon material was donated as xDMC-T, where x stands for the initial amount of DCDA, and T stands for post-carbonization temperature. The pure carbon sample with post-carbonization at 550 °C were donated as MC-550. The morphology of the nitrogen-doped carbon materials was first investigated by SEM and TEM measurements. Both SEM and TEM analysis of nitrogen-doped samples displayed the hierarchical porous structure. As shown in Fig. 1, SEM image of both the MC-550 (Figure 1a) and 0.4DMC-550 sample (Figure 1b) exhibited well-dispersed spherules structure. Figure 1c–i showed TEM images of nitrogen-doped and undoped carbon samples. The TEM analysis also revealed the presence of small spherical nanoparticles with nanoporous structure in the MC-550 sample (Figure 1c). The addition of small amount of DCDA would lead to a slight increase in primary particle size. The 0.5DMC-550 sample (Figure 1d) retained small nanoparticles. Excessive DCDA would cause a sharp rise in the particle size. When the mass of DCDA was increased to 0.8 g, the sample showed a layered structure with few pores, which suggested that excessive DCDA might hamper the formation of nanoporous structure.

**Figure 2:**
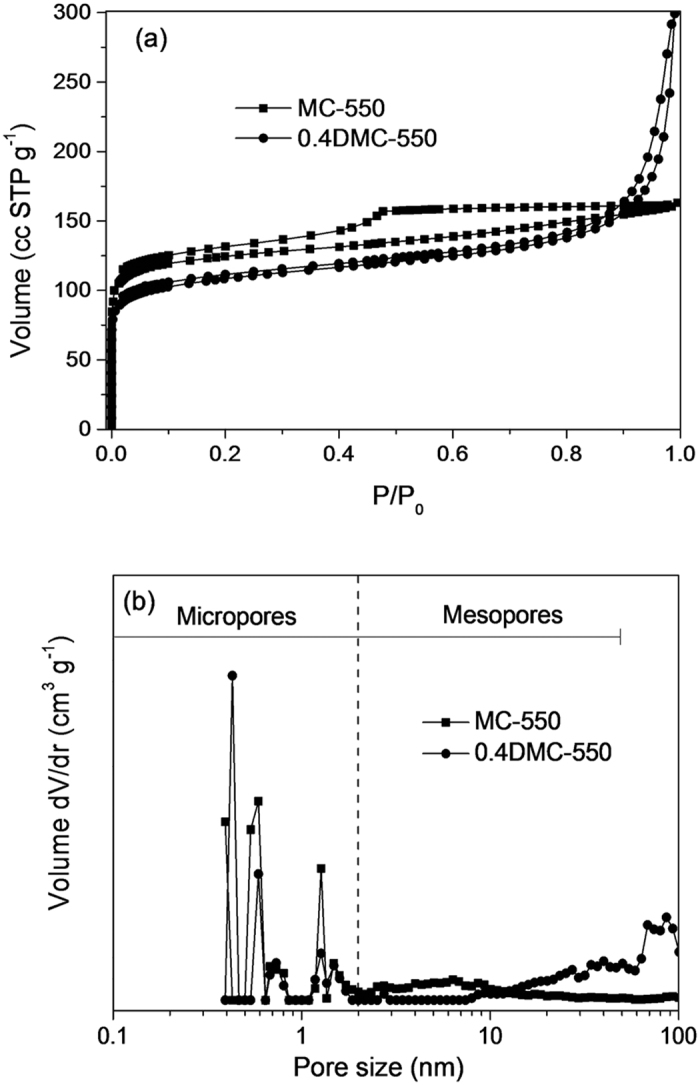
(**a**) Nitrogen adsorption/desorption isotherms of MC-550 and 0.4DMC-550, (**b**) pore-size distribution curves of MC-550 and 0.4DMC-550.

**Figure 3:**
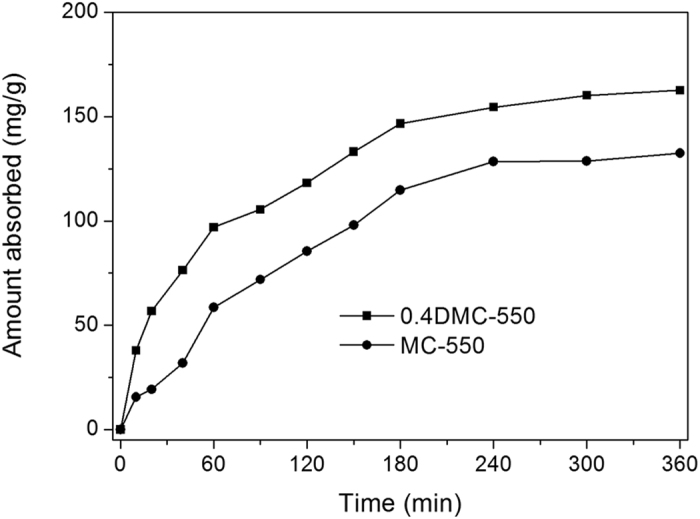
The amount of butanol absorbed on MC-550 and 0.4DMC-550.

Nitrogen sorption measurement allowed quantification of the interstitial porous system. Figure 2a showed the adsorption/ desorption isotherms of the MC-550 and 0.4DMC-550 samples. Both the carbon samples displayed considerable N_2_ adsorption at low pressure of 0-0.1, reflecting the existence of microporosity. The MC-550 showed type-IV isotherm with H1-type hysteresis loop, indicating the occurrence of mesoporous structure. 0.4DMC-550 exhibited type-IV curves with H3 hysteresis loop and a sharp steep at the relative high pressure of ~0.8, reflecting a large external surface area and the presence of excessive mesoporosity. Figure 2b gave the pore-size distribution curves of MC-550 and 0.4DMC-550. Both the samples contained a similar fraction of micropores with a size around 0.5 nm and 1.5 nm. The MC-550 showed mesoporous structure with the pore size in the range of 2–13 nm, and the mesopore size of DMC-500 enlarged markedly. The calculated BET surface areas and pore volume of MC-550 and 0.4DMC-550 were 510 m^2^ g^−1^ and 470 m^2^ g^−1^, 0.25 cm^3^ g^−1^ and 0.46 cm^3^ g^−1^, respectively. The results showed that although the surface area declined slightly after the introduction of nitrogen functionalities, the pore volume increased drastically. The porosity of MC-550 sample was made up of 65 vol% of microporosity and 35 vol% of mesoporosity (V_micro_~0.16 cm^3^ g^−1^, V_meso_~0.09 cm^3^ g^−1^). Nitrogen functionalities did not increase the micropore content (V_micro_~0.15 cm^3^ g^−1^ for 0.4DMC-550), but significantly increased the mesopore content from 0.09 cm^3^ g^−1^ to 0.41 cm^3^ g^−1^. The results suggested that a proper amount of DCDA addition would facilitate the formation of large-sized mesopores in the carbons. The formation of large mesopores was speculated to be in relation to the high nitrogen content of DCDA precursor, which generated gaseous species (e.g. N_2_ and NH_3_) during the carbonizing process. The decomposition of the DCDA and the escape of these gas from the carbon might result in the formation of the large-sized mesopores. A similar effect was also observed by Zhao *et al.*^33^ and Yao *et al.*^34^. In addition, the mesoporosity of nitrogen-doped carbon materials also might partly originate from the interparticle pores between primary carbon particles. The carbon materials with well hierarchical porous structure and nitrogen functionality would be interesting supports for the immobilization of small-sized noble metal. Moreover, heterogeneous catalysis was known to be a surface-based process. The meso/microporous structures of materials would not only provide them with high BET surface area and stronger surface adsorption ability to the reactant molecules, but also be beneficial to enhance the diffusion of the reactant molecules during catalytic reaction. For the nitrogen-doped samples with proper amount of DCDA addition, both the incorporated nitrogen atoms and the increased mesostructure would be favourable for the adsorption of the substrates on the surface of the catalyst, which in principle could boost the catalytic process. The conjecture was supported by our experimental results of the butanol adsorption over 0.4DMC-550 and MC-550 samples. As shown in the Figure 3, after adsorption for 6 h, the butanol adsorbed over 0.4DMC-550 (163 mg/g) was higher than that on MC-550 (132 mg/g).

The surface area and the chemical composition of all the nitrogen-doped carbon samples with different addition DCDA amounts and different post-carbonization temperatures were summarized in Table 1. Elemental analysis showed that with increasing addition of DCDA, the incorporated nitrogen content rose from 0 to 9.8 wt% for nitrogen-doped carbon samples. From the N_2_ sorption analysis, we can see that MC-550 had a high surface area of 510 m^2^ g^−1^. With the increasing DCDA addition amount to 0.7 g, the surface area of DMC-550 samples reduced gradually from 510 m^2^ g^−1^ to 324 m^2^ g^−1^, indicating that the porous structures can well retained in the range. When the DCDA amount was further increased to 0.8 g, the surface area dropped sharply to 20 m^2^ g^−1^, which was also confirmed by the layered structure with few pores in the TEM image. The results suggested that the increasing addition of DCDA could result in the raising content of incorporated nitrogen in the carbon materials, while excessive DCDA would destroy porous structure. Thus, the optimized amount of DCDA addition was 0.4-0.5 g, where the samples well maintained nanoporous structure and had relatively high nitrogen content. The textural and chemical properties were also affected by the post-carbonization temperature. The incorporated nitrogen content reduced from 5.8 wt% to 3.4 wt% for 0.4DMC with the rise of the carbonization temperature from 550 ^o^C to 900 ^o^C, while the surface area showed a slight uptrend from 470 m^2^ g^−1^ to 509 m^2^ g^−1^. The result revealed that some nitrogen species was removed by reaction with oxygenated functions or formation of gaseous products (e.g. N_2_ and NH_3_) during the post-carbonization process at the high temperature.

**Figure 4:**
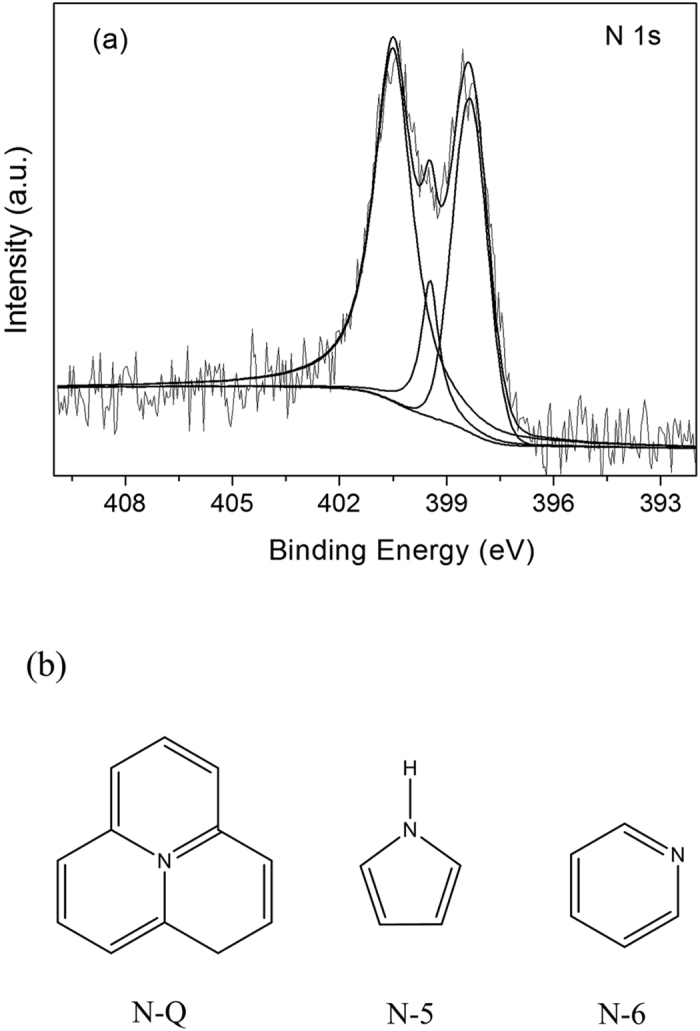
(**a**) N1s XPS spectra of 0.4DMC-550 sample, (**b**) types of nitrogen functionalities.

**Figure 5:**
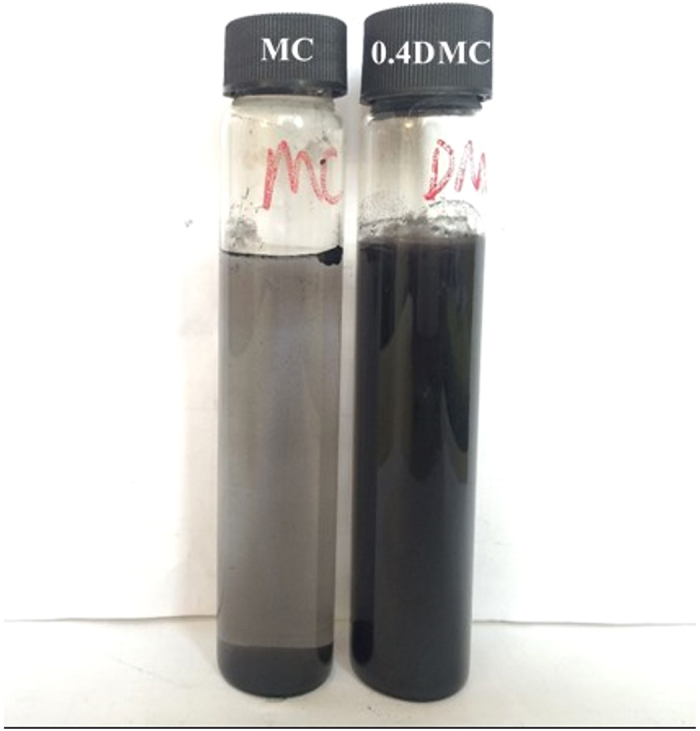
Photographs of the dispersions of 0.4DMC-550 and MC-550 in water, after sonication for 5 min and storage for 10 min under ambient conditions.

The nature of the nitrogen species at the surface of the porous nitrogen-containing carbon sample was analyzed by the high-resolution XPS measurement. The XPS spectra of N 1s of 0.4DMC-550 material was shown in Figure 4. Three peaks at 400.5 eV, 399.5 eV and 398.4 eV could be fitted, ascribed to quaternary nitrogen (N-Q), pyrrolic nitrogen (N-5) and pyridinic nitrogen (N-6), respectively^35^,^36^. The results suggested that the nitrogen elements were indeed incorporated into the carbon skeletons. The incorporated nitrogen content calculated by the XPS measurement was about 5.9%, which was very similar to the elemental analysis result of 5.8%. The results supported that nitrogen atoms were homogeneously incorporated in the framework of the carbon materials. The detailed results of curve-fitting of the N 1s spectra showed that the proportions of N-Q, N-5 and N-6 were 33.7%, 10.4% and 55.9%, respectively. As pyridinic nitrogen can behave both as Lewis and Brönsted base, the high content of pyridinic nitrogen implied the presence of abundant basic functional sites in the nitrogen-doped samples^32^,^37^. Moreover, nitrogen incorporation would make the carbon materials better dispersibility in water. As shown in Figure 5, when the 0.4DMC-550 and MC-550 was suspended in water, followed by sonicated for 5 min, nitrogen-doped carbon sample was well dispersed in water after storage for 10 min under ambient conditions, and pure carbon sample was easily to precipitate.

**Figure 6:**
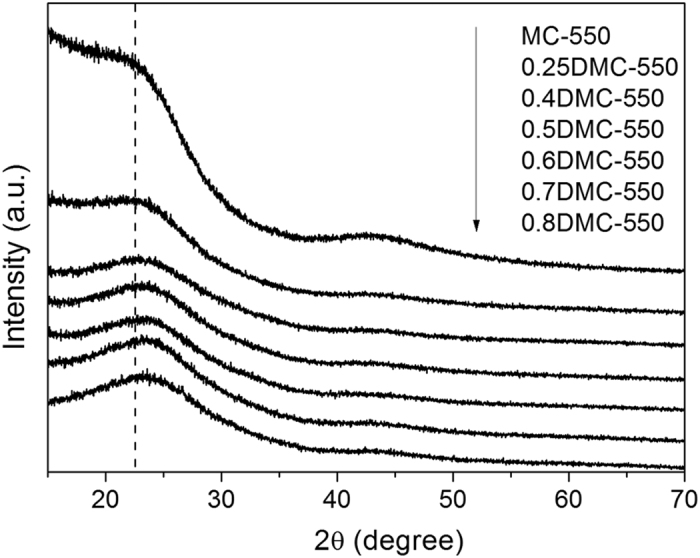
XRD patterns of the as-made carbon samples.

The effect of nitrogen functionality on the structure of carbon materials was further examined by XRD. Figure 6 displayed the XRD patterns of undoped and nitrogen-doped carbon samples. Two characteristic broad and weak peaks could be found at around 23° and 44° for all the samples, corresponding to the graphitic carbon (002) and (100) crystal planes, respectively^38^. The broad and weak peaks indicated the low degree of graphitization of carbon samples. A careful observation showed that the (002) peak of the nitrogen-doped carbon samples was shifted to high angle, compared to the undoped carbon sample. This observation suggested the d- space of (002) crystal plane in the graphitic structure was reduced after nitrogen functionality, indicating that nitrogen incorporation in the carbon samples resulted in more compact graphitic structures.

### Characterizations of the Ir catalysts

**Figure 7:**
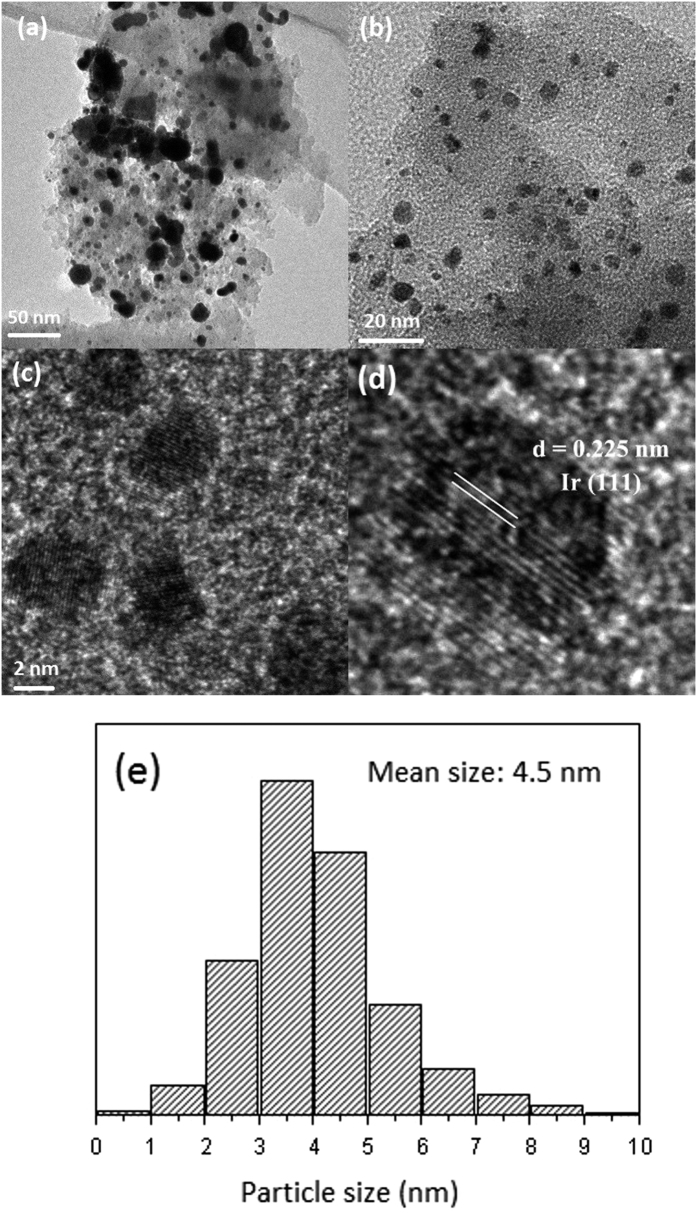
TEM images of (**a**) 5%Ir-MC-550 and (**b**) 5%Ir-0.4DMC-550 samples. HRTEM images of (**c,d**) 5%Ir-0.4DMC-550, (**e**) Ir particle size distribution of 5%Ir-0.4DMC-550 sample.

Ir-DMC catalysts with different Ir loadings (0–5 wt%) were synthesized by the impregnation method. TEM was used to study the morphology and particle size of 5%Ir-0.4DMC-550 and 5%Ir-MC-550 catalysts. Representative TEM micrographs were presented in Figure 7. For the 5%Ir-0.4DMC-550 sample, the Ir nanoparticles with an average size of 4.5 nm were uniformly dispersed on the nitrogen-doped carbon materials. HRTEM images of the 5%Ir-0.4DMC-550 given in Figure 7c,d were indicative of the highly crystalline feature of the Ir nanoparticles, which clearly showed an interplanar spacing of 0.225 nm, attributable to the {1 1 1} plane of face centered cubic Ir. For the comparison, the Ir loaded on MC-550 without nitrogen functionality was also measured (shown in Figure 7a). A large cluster of multiple Ir particles on MC-550 can be clearly observed in the TEM images, suggesting that Ir particles could not be well dispersed on pure carbon support. The aggregation of Ir nanoparticles might be caused by the chemical inertness of the undoped carbon materials. The results reflected that the immobilization and dispersion of the Ir nanoparticles on the carbon supports could be improved by the nitrogen functionality. Compared with the supports, the as-made Ir-DMC catalysts (Table 2) afforded similar porous nature with high surface area (440 m^2^ g^−1^) and pore volume (0.46 cm^3^ g^−1^). It is believed that the well maintained hierarchical porous structure and abundant functional sites of the nitrogen-doped materials could result in the formation of a strong interaction between support surface and Ir nanoparticles, giving rise to the uniform deposition of noble metal nanoparticles.

**Figure 8:**
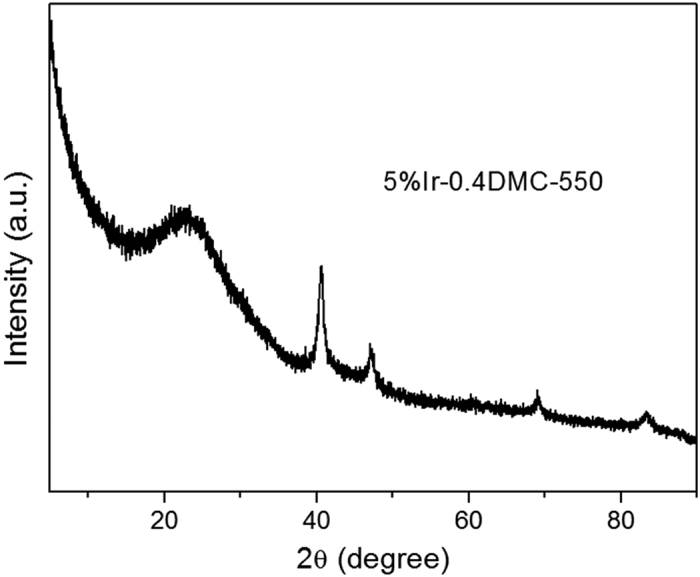
XRD patterns of 5%Ir-0.4DMC-550 samples.

**Figure 9:**
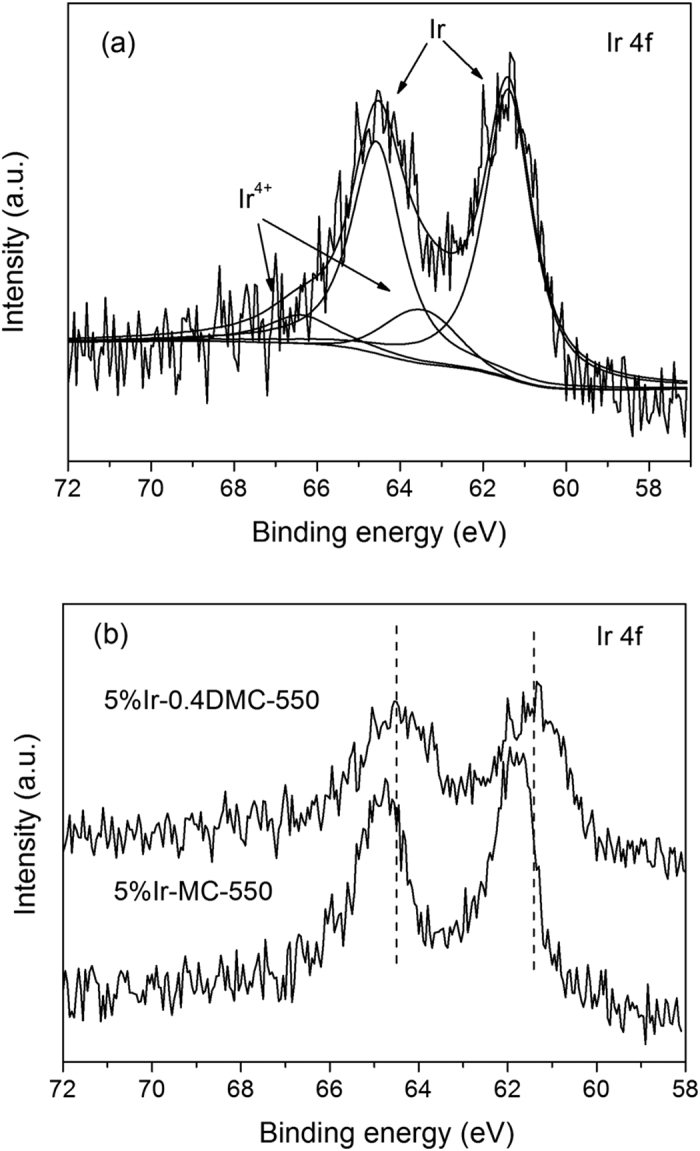
(**a**) Ir 4f XPS spectra of 5%Ir-0.4DMC-550 sample, (**b**) Ir 4f XPS spectra of 5%Ir-0.4DMC-550 and 5%Ir-MC-550 sample.

The 5%Ir-0.4DMC-550 sample was further characterized by XRD and XPS to obtain the structural information. As shown in Figure 8, the diffraction peaks at 40.7°, 47.3°, 69.2°, 83.4° and 88.1° were all observed in 5%Ir-0.4DMC-550, attributed to (111), (200), (220), (311) and (222) peaks of metallic Ir^0^ 39. The 5%Ir-DMC-500 showed a broad (111) peak and a low intensity. The crystallite sizes of the iridium particles, estimated from Scherrer’s equation by (111) peak, were about 5 nm for 5%Ir-0.4DMC-550. The result was in good agreement with TEM result, supporting that Ir nanoparticles could be well dispersed on the surface of DMC-550. The XPS analysis of the Ir 4f for 5%Ir-0.4DMC-550 was shown in Figure 9a. The Ir 4f spectra can be fitted into two doublets. The peaks at 61.4 and 64.5 eV for Ir 4f _7/2_ and Ir 4f _5/2_ were assigned to metallic Ir^0^ 40, while the peaks at 63.5 and 66.4 eV were characteristic of Ir^4+^ 41. The atom ratios of Ir^0^ and Ir^4+^ calculated by XPS results were 83.6% and 16.4%, respectively. The results suggested that the Ir species on the surface of nitrogen doped carbon materials were mainly in the metallic state. The small amount of Ir^4+^ species may be resulted from the incomplete reduction of the Ir species during the calcination under an inert N_2_ atmosphere. Figure 9b showed the XPS spectra of Ir 4f of 5%Ir-0.4DMC-550 and 5%Ir-MC-550. Compared to 5%Ir-MC-550 sample, the Ir 4f peaks of 5%Ir-0.4DMC-550 was shifted to a lower binding energy. This small shift toward low energy indicated that the electron density of the Ir atom was enhanced by transferring the electron from nitrogen doped supports to Ir, which would further strengthen the interaction between Ir and support.

### Self-condensation of butanol with Ir catalysts in aqueous phase

**Figure 10:**
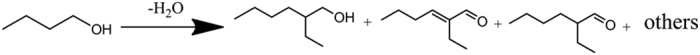
Main products in butanol condensation.

Butanol, as a typical bio-alcohol, is a main raw materials to produce biological and medicinal chemicals^42^. Direct self-condensation of butanol to 2-ethylhexanol in one pot under mild condition is a promising alternative to current industrial production of 2-ethylhexanol with three reaction steps^43^. Here, the self-condensation of butanol in aqueous phase was chosen as the model reaction to evaluate the catalytic activity of the carbon supported Ir samples. The results were displayed in Table 3. The products of butanol condensation were mainly 2-ethylhexanol, 2-ethylhexanal and 2-ethyl-2-hexenal (shown in Figure 10). The mass balance of butanol and detected products was >95%. 5%Ir-MC-550 showed very poor activity for the butanol condensation in aqueous phase (Entry 2, Table 3). When the carbon support was modified with nitrogen, significant improvement of desired 2-ethylhexanol yield could be achieved. With 5%Ir-0.25DMC-550 catalyst, the condensation afforded a 34.5% 2-ethylhexanol yield based on butanol within 17 h (Entry 3, Table 3), which was also accompanied by the formation of 2-ethylhexanal (1.1%) and 2-ethyl-2-hexenal (<0.1%). The activity can be further enhanced with the increasing of DCDA addition amount, and the 5%Ir-0.4DMC-550 catalyst had the highest 2-ethylhexanol yield of 39.9% (Entry 4, Table 3) with 96.7% 2-ethylhexanol selectivity. However, higher DCDA addition amount than 0.4 g led to a decreased activity (Entry 5–7, Table 3). The results indicated that a suitable DCDA addition amount is crucial for optimizing the activity of Ir catalysts. Although the increased DCDA addition amount led to an increase in the incorporated nitrogen content, excessive DCDA would also destroy the porous structure of carbon materials, which had negative effect on the catalytic process.

The impact of the post-carbonization temperature on the self-condensation of butanol was also investigated. With the elevated post-carbonization temperature from 550 ^o^C to 700 ^o^C, the butanol conversion was improved from 41.2% to 49.8%, but the 2-ethylhexanol selectivity was declined from 96.7% to 93.2% (Entry 4, 8, Table 3). Even higher temperature would suppress the activity with little difference of 2-ethylhexanol selectivity, probably due to the loss of nitrogen species on the surface of the supports (Entry 9–10, Table 3). The obvious differences in the activities demonstrated that the incorporated nitrogen in the carbon support played an essential role in the formation of catalytically active sites and creation of an efficient catalyst. The result was in agreement with the earlier observation reported by Wang^32^ and Xiong *et al.*^44^. Recently, NH and NH_2_ groups on the surface of nitrogen-rich carbon materials were demonstrated to effectively adsorb alcohols such as phenol, benzyl alcohol by O…H-N or O-H…N interaction^32^,^45^,^46^. As the surface of the doped carbon samples had abundant basic functional groups (C–N, N-H etc.), butanol could be more easily adsorbed on the surface of basic support by the formation of O-H…N interactions, which was confirmed by the experimental results of the butanol adsorption (see Figure 3).

The activity was also affected by the reaction temperature. When 5%Ir-0.4DMC-550 was used as catalyst, with the elevated temperature from 150 ^o^C to 190 ^o^C, the butanol conversion was enhanced evidently from 31.7% to 55.3%, but highest 2-ethylhexanol selectivity was obtained at 170 ^o^C (Entry 4, 11-12, Table 3). The effect of Ir loading on the catalytic activity was also studied in the similar condition (Entry 4, 13-14, Table 3). Compared with the 5% Ir-0.4DMC-550 catalyst (TOF: 19 h^−1^), 1% Ir-0.4DMC-550 showed three times higher TOF of 63 h^−1^ with high 2-ethylhexanol selectivity of ~97%. Undoubtedly, the convenient separation, recovering and reusing of catalyst were the best merit factors for the heterogeneous catalysis. The capability of recycling the novel Ir-based catalysts was studied by the self-condensation of butanol at 170 ^o^C after washed and dried. The results (Entry 4, 15–18, Table 3) showed that the catalytic activities of the four consecutive runs were similar to that of fresh 5%Ir-0.4DMC-550, demonstrating the sufficient stability of nitrogen-doped carbon supported Ir catalyst in the butanol condensation in water. The reactant mixture after reaction was analyzed by AAS. Element analysis showed that with 5%Ir-DMC-550 catalyst, no dissolved Ir was detected. The color of the aqueous solvent after reaction also maintained colorless, reflecting the high stability of 5%Ir-DMC-550 catalyst. When 5%Ir-MC-550 catalyst was used as the catalyst, about 10% iridium in 5%Ir-MC-550 might leach into the aqueous solvent. The result indicated that nitrogen-enriched carbon surface made the interaction between iridium and carbon support much tighter. All these results suggested that the butanol condensation in water was indeed promoted by heterogeneous catalysis, and the nitrogen-doped carbon supported Ir catalysts were water tolerant, high active and stable in water system.

Some control experiments were also carried out with 5%Ir-DMC-550 catalyst to study the active metal. 5%Ir-0.4DMC-550 was further reduced at 450 ^o^C for 4 h under H_2_ atmosphere, and oxidized at 250 ^o^C for 4 h under air atmosphere, respectively. It can be observed that when 5%Ir-DMC-550 was reduced under H_2_ atmosphere, the butanol conversion and 2-ethylhexanol yield slightly improved from 41.2% to 45.5%, and from 39.9% to 42.8%, respectively; while further oxidation of 5%Ir-DMC-550 sample under air atmosphere greatly suppressed the activity with low conversion of 19.6% and 2-ethylhexanol yield of 14.7%. Combined with the results that Ir species on the surface of 5%Ir-0.4DMC-550 were also mainly in the metallic state (the atom ratios of Ir^0^ and Ir^4+^ were 83.6% and 16.4%), it could be concluded that Ir^0^ was more effective for butanol conversion and 2-ethylhexanol selectivity.

Based on these characterized results above, the improved catalytic activity of nitrogen-doped carbon based catalysts could be attributed to several factors. Firstly, nitrogen-doped materials have good dispersibility in water, which is favourable for the catalytic reaction in aqueous phase. Secondly, the incorporated nitrogen could facilitate the highly dispersion and stabilization of Ir nanoparticles on the surface of carbon supports. The electron-rich nitrogen atoms would interact with Ir nanoparticles more preferably than carbons by electron donation from the nitrogen atom to the Ir, leading to a higher electron density on the Ir surface (supported by XPS analysis). The stronger interaction between Ir particles and nitrogen species on the surface of carbon support might afford catalytically active sites for butanol condensation. Thirdly, the incorporated nitrogen might interact with butanol substrates by O-H…N interaction and the nitrogen-doped catalysts would adsorb the substrates more efficiently than those without nitrogen functionality. The stronger butanol adsorption ability of nitrogen-doped catalysts also contributed to the increased activity.

## Conclusions

Based on these results above, Ir nanoparticles supported on nitrogen-doped porous carbons were demonstrated as a kind of efficient heterogeneous catalysts for the self-condensation of bio-alcohols in aqueous phase. The nitrogen-doped carbon materials were produced by HTC method with DCDA as nitrogen source. During the hydrothermal carbonization, nitrogen-containing functional groups (C–N, N-H etc.) were introduced to the edges and onto the basal plane of the porous carbon material. The contents of incorporated nitrogen were varied from 0 to 9 wt% by adjusting the DCDA addition amount. The nitrogen-doped carbons possessed high surface area composed of micropores and large continuous mesopores with excellent hierarchical nanostructure. These excellent properties made the nanostructured doped materials ideal support for immobilizing Ir nanoparticles. The nitrogen doping not only led to well dispersion and stabilization of Ir nanoparticles, but also enhanced the absorbance of butanol substrates and increased the catalytically active sites, finally improved the activity for the self-condensation of butanol. Catalytic studies showed that the novel catalysts exhibited excellent catalytic activity in butanol condensation in water with high 2-ethylhexanol selectivity. What’s more, the Ir-based catalysts showed highly stability and reusability in the butanol condensation in aqueous phase.

## Methods

### Synthesis of nitrogen-doped carbon materials

The nitrogen-doped carbon material was synthesized using a HTC method^32^. In a typical process, a mixture containing 9 g glucose, 0.75 g borax, certain amount of DCDA (0–1.2 g) and 20 mL deionized water were stirred for 2 h. Then, these mixed solution was moved to a Teflon-lined autoclave and kept at 200 °C for 8 h. The obtained solids were then filtered, washed and dried. The materials were heated at 550, 700, 800 and 900 °C for 4 h under N_2_ and cooled to the ambient temperature naturally.

### Synthesis of Ir@N-doped carbon materials

IrCl_3_ (1 wt%, 5 mg Ir; 3 wt%, 15 mg Ir; 5 wt%, 25 mg Ir) dissolved in deionized water was mixed with 500 mg xDMC-T. This resultant mixture was stirred at room temperature for overnight and dried at 100 ^o^C to remove water. Then, it was calcined at 550 °C for 4 h under an inert N_2_ atmosphere to produce the final Ir-based samples. The supported Ir samples were denoted as y%Ir-xDMC-T, where y stands for the amount of Ir.

### Characterizations

X-ray powder diffraction (XRD) patterns were taken by a Bruker D8 Advanced X-ray diffractometer using CuKa radiation (λ = 1.5147 Å). Nitrogen adsorption measurements were performed at 77 K by a micromeritics ASAP 2020 m + c sorptometer with nitrogen gas as the sorbate. All the samples were heated at 160 ^o^C for 10 h under vacuum before analysis. The morphologies of the as-prepared samples were measured by a field emission Hitachi S-4800 scanning electron microscope (SEM) and a H-7600 transmission electron microscopy (TEM). High resolution TEM images were collected by a field emission Tecnai G2F20 electron microscope. Elemental composition of the nitrogen-doped carbons was analyzed using a Vario El elemental analyzer. The X-ray photoelectron spectroscopy (XPS) analysis were conducted using a Thermo ESCALAB250 instrument with a monochromatized AlKa line source. The binding energies of the samples were referenced to C1s peak being at 284.6 eV. The leached Ir in solution was analyzed by an INESA 4510F atomic absorption spectrometer (AAS).

### Butanol adsorption

In each experiment, 200 mg of carbon material was suspended in aqueous solution of butanol (100 mL, 1000 ppm), which was then stirred at 20 ^o^C. At a given time, the solution (1.5 mL) was sampled, centrifuged and added with methanol (volume 1:1). The butanol concentration was measured by a gas chromatograph (VARIAN 450). The amount of butanol adsorption was calculated from the following: the amount of butanol adsorption = 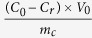. Here C_0_ and C_r_ stands for the concentration of butanol in the aqueous solution at initial and residual butanol concentration after adsorption, V_o_ is the volume of aqueous solution, m_c_ is the mass of carbon.

### Catalytic activity for butanol condensation

The activity of the catalysts was evaluated by the self-condensation of butanol in water. In a typical reaction, 1 g butanol, 0.1 g catalyst, 1 equiv. of NaOH and 15 mL deionized water were added into a 50 mL Teflon-lined autoclave. The reactions were then heated to the desired temperature (150–190 ^o^C) with magnetic stirring. At the end of the reaction, ether was used to extract the products. The products in aqueous and organic phase were then analyzed by a GC (VARIAN 450) and GC–MS^18^.

## Additional Information

**How to cite this article**: Liu, D. *et al.* Iridium nanoparticles supported on hierarchical porous N-doped carbon: an efficient water-tolerant catalyst for bio-alcohol condensation in water. *Sci. Rep.* **6**, 21365; doi: 10.1038/srep21365 (2016).

## Figures and Tables

**Table 1:** Compositions of the carbon materials and their surface areas.

Sample	C^a^ (%)	N^a^ (%)	N/C (at%)	S_BET_^b^ (m^2^ g^−1^)
MC-550	–	–	–	510
0.25DMC-550	86.7	3.8	0.044	490
0.4DMC-550	87.1	5.8	0.066	470
0.5DMC-550	82.0	6.5	0.080	435
0.6DMC-550	82.0	6.7	0.082	370
0.7DMC-550	79.6	7.0	0.087	324
0.8DMC-550	79.4	7.5	0.094	20
1.2DMC-550	78.5	9.8	0.124	2
0.4DMC-700	86.3	4.6	0.053	475
0.4DMC-800	89.76	4.5	0.050	496
0.4DMC-900	90.33	3.4	0.038	509

^a^Derived from elemental CHN analysis.

^b^BET Surface area.

**Table 2:** Textural properties of MC-500, 0.4DMC-550 and the Ir catalysts.

Sample	S_BET_ (m^2^ g^−1^)	V^a^_total_ (cm^3^ g^−1^)	V^b^_micro_ (cm^3 −1^)	V^c^_meso_ (cm^3^ g^−1^)
MC-550	510	0.25	0.16	0.09
0.4DMC-550	470	0.56	0.15	0.41
5%Ir-MC-550	490	0.23	0.15	0.08
5%Ir-0.4DMC-550	440	0.46	0.14	0.32

**Table 3:** Self-condensation of butanol catalyzed by Ir catalysts in aqueous phase^a^.

Entry	Catalyst	T (°C)	Conv. (%)	Yield (Sele.) of2-ethylhexanol (%)	TOF^b^ (h^−1^)
1	–	170	–	–	–
2	5%Ir-MC-550	170	3.2	1	1
3	5%Ir-0.25DMC-550	170	37.8	34.5 (83.1)	17
4	5%Ir-0.4DMC-550	170	41.2	39.9 (96.7)	19
5	5%Ir-0.5DMC-550	170	28.2	27.2 (96.5)	13
6	5%Ir-0.6DMC-550	170	19	16.9 (88.5)	9
7	5%Ir-0.7DMC-550	170	18	16.5 (91.5)	8
8	5%Ir-0.4DMC-700	170	49.8	46.5 (93.2)	23
9	5%Ir-0.4DMC-800	170	40.8	38.4 (94.1)	19
10	5%Ir-0.4DMC-900	170	32.6	30.7 (94.3)	15
11	5%Ir-0.4DMC-550	150	31.7	27.3 (86.1)	15
12	5%Ir-0.4DMC-550	190	55.3	42.8 (77.3)	25
13	1%Ir-0.4DMC-550	170	27.5	27 (98)	63
14	3%Ir-0.4DMC-550	170	44.9	43.6 (97.1)	34
15^c^	Reuse entry 4 for 2^nd^	170	41.8	40.2 (96.2)	19
16^c^	Reuse entry 4 for 3^rd^	170	40.2	38.9 (96.9)	18
17^c^	Reuse entry 4 for 4^th^	170	40.7	39.2 (96.3)	19
18^c^	Reuse entry 4 for 5^th^	170	39.6	37.9 (95.6)	18

^a^Reaction conditions: 1 g butanol, 0.1 g iridium catalysts, 1.0 equiv. of NaOH, 15 ml H_2_O, 17 h.

^b^TOF = [reacted mol butanol]/[(total mol metal) * (reacton time)].

^c^The catalysts were washed with ethanol and water, and then dried for the recycling test.

## References

[b1] Tsuchida T., Sakuma S., Takeguchi T. & Ueda W. Direct synthesis of n-butanol from ethanol over nonstoichiometric hydroxyapatite. Ind. Eng. Chem. Res. 45, 8634–8642 (2006).

[b2] Leon M., Diaz E., Vega A., Ordonez S. & Auroux A. Consequences of the iron–aluminium exchange on the performance of hydrotalcite–derived mixed oxides for ethanol condensation. Appl. Catal. B 102, 590–599 (2011).

[b3] Di Cosimoa J. I., Apestegui C. R., Ginesb M. J. L. & Iglesiab E. Structural requirements and reaction pathways in condensation reactions of alcohols on Mg_*y*_AlO_*x*_ catalysts. J. Catal. 190, 261–275 (2000).

[b4] Gines M. J. L. & Gines E. Bifunctional condensation reactions of alcohols on basic oxides modified by copper and potassium. J. Catal. 176, 155–172 (1998).

[b5] Hilmen A. M., Xu M. T., Gines M. J. L. & Iglesia E. Synthesis of higher alcohols on copper catalysts supported on alkali–promoted basic oxides. Appl. Catal. A 169, 355–372 (1998).

[b6] Kozlowski J. T. & Davis R. J. Heterogeneous catalysts for the guerbet coupling of alcohols. ACS Catal. 3, 1588–1600 (2013).

[b7] Gunanathan C. & Milstein D. Applications of acceptorless dehydrogenation and related transformations in chemical synthesis. Science 341, 249–261 (2013). 10.1126/science.1229712 23869021

[b8] Sheldon R. A. Recent advances in green catalytic oxidations of alcohols in aqueous media. Catal. Today 247, 4–13 (2015).

[b9] Farsani M. R., Assady E., Jalilian F., Yadollahi B. & Rudbari H. A. Green oxidation of alcohols with hydrogen peroxide catalyzed by a tetra–cobalt polyoxometalate in water. J. Iran. Chem. Soc. 12, 1207–1212 (2015).

[b10] Matsu-ura T., Sakaguchi S., Obora Y. & Ishii Y. Guerbet reaction of primary alcohols leading to β-alkylated dimer alcohols catalyzed by iridium complexes. J. Org. Chem. 71, 8306–8308 (2006). 17025333 10.1021/jo061400t

[b11] Gregorio G., Pregaglia G. F. & Ugo R. Condensation of alcohols catalysed by tertiary phosphine transition metal complexes. J. Org. Chem. 37, 385–387 (1972).

[b12] Burk P. L., Pruett R. L. & Campo K. S. The rhodium-promoted guerbet reaction: part II. secondary alcohols and methanol as substrates. J. Mol. Catal. 33, 15–21 (1985).

[b13] Koda K., Matsu-ura T., Obora Y. & Ishii Y. Guerbet reaction of ethanol to n-butanol catalyzed by iridium complexes. Chem. Lett. 38, 838–839 (2009).

[b14] Carlini C., Macinai A., Galletti A. M. R. & Sbrana G. Selective synthesis of 2-ethyl-1-hexanol from n-butanol through the guerbet reaction by using bifunctional catalysts based on copper or palladium precursors and dodium butoxide. J. Mol. Catal. A 212, 65–70 (2004).

[b15] Marcu I. C., Tanchoux N., Fajula F. & Tichit D. Catalytic conversion of ethanol into butanol over M–Mg–Al mixed oxide catalysts (M = Pd, Ag, Mn, Fe, Cu, Sm, Yb) obtained from LDH precursors. Catal. Lett. 143, 23–30 (2013).

[b16] Carvalho D. L., Avillez R. R., Rodrigues M. T., Borges L. E. P. & Appel L. G. Mg and Al mixed oxides and the synthesis of n-butanol from ethanol. Appl. Catal. A 415–416, 96–100 (2012).

[b17] Tanaka Y. & Utsunomiya M., inventors; Mitsubishi Chemical Corp., assignee. Process of producing alcohol. *United States patent US* 8,318,990. 2012 Nov 27.

[b18] Xu G. Q. *et al.* Direct self-condensation of bio-alcohols in the aqueous phase. Green Chem. 16, 3971–3977 (2014).

[b19] Liang C. D., Li Z. J. & Dai S. Mesoporous carbon materials: synthesis and modification. Angew. Chem. Int. Ed. 47, 3696–3717 (2008). 10.1002/anie.200702046 18350530

[b20] Yang Z. H. & Nakashima N. A simple preparation of very high methanol tolerant cathode electrocatalyst for direct methanol fuel cell based on polymer-coated carbon nanotube/platinum. Sci. Rep. 5, 12236 (2015) 26192397 10.1038/srep12236 PMC4507447

[b21] Fellinger T. P., White R. J., Titirici M. M. & Antonietti M. Borax-mediated formation of carbon aerogels from glucose. Adv. Funct. Mater. 22, 3254–3260 (2012).

[b22] Nieto-Márquez A., Toledano D., Sánchez P., Romero A. & Valverde J. L. Impact of nitrogen doping of carbon nanospheres on the nickel–catalyzed hydrogenation of butyronitrile. J. Catal. 269, 242–251 (2010).

[b23] Paraknowitsch J. P., Thomas A. & Antonietti M. A detailed view on the polycondensation of ionic liquid monomers towards nitrogen doped carbon materials. J. Mater. Chem. 20, 6746–6758 (2010).

[b24] Xu X. *et al.* Synthesis of palladium nanoparticles supported on mesoporous N-doped carbon and their catalytic ability for biofuel upgrade. J. Am. Chem. Soc. 134, 16987–16990 (2012). 23030399 10.1021/ja308139s

[b25] Ayusheev A. B. *et al.* Ruthenium nanoparticles supported on nitrogen-doped carbon nanofibers for the catalytic wet air oxidation of phenol. Appl. Catal. B 146, 177–185 (2014).

[b26] Jiang S. J. *et al.* Effect of doping the nitrogen into carbon nanotubes on the activity of NiO catalysts for the oxidation removal of toluene. Appl. Catal. B 160–161, 716–721 (2014).

[b27] Hu B. *et al.* Engineering carbon materials from the hydrothermal carbonization process of biomass. Adv. Mater. 22, 813–828 (2010). 20217791 10.1002/adma.200902812

[b28] Zhai Y. *et al.* Carbon materials for chemical capacitive energy storage. Adv. Mater. 23, 4828–4850 (2011). 21953940 10.1002/adma.201100984

[b29] Wang H. B., Maiyalagan T. & Wang X. Review on recent progress in nitrogen-doped graphene: synthesis, characterization, and its potential applications. ACS Catal. 2, 781–794 (2012).

[b30] Sheng X. *et al.* N-doped ordered mesoporous carbons prepared by a two-step nanocasting strategy as highly active and selective electrocatalysts for the reduction of O_2_ to H_2_O_2_. Appl. Catal. B 176–177, 212–224 (2015).

[b31] Lim K. H. & Kim H. S. Nitrogen-doped carbon catalysts derived from ionic liquids in the presence of transition metals for the oxygen reduction reaction. Appl. Catal. B 158–159, 355–360 (2014).

[b32] Zhang P. F., Gong Y. T., Li H. R., Chen Z. R. & Wang Y. Solvent-free aerobic oxidation of hydrocarbons and alcohols with Pd@N-doped carbon from glucose. Nat. Commun. 4, 1593–1604 (2013). 23481401 10.1038/ncomms2586

[b33] Wei J. *et al.* A controllable synthesis of rich nitrogen-doped ordered mesoporous carbon for CO_2_ capture and supercapacitors. Adv. Funct. Mater. 23, 2322–2328 (2013).

[b34] Zhang H. *et al.* Porous nitrogen doped carbon foam with excellent resilience for self-supported oxygen reduction catalyst. Carbon 95, 388–395 (2015)

[b35] Niu W. H. *et al.* Mesoporous N-doped carbons prepared with thermally removable nanoparticle templates: an efficient electrocatalyst for oxygen reduction reaction. J. Am. Chem. Soc. 137, 5555–5562 (2015). 25860843 10.1021/jacs.5b02027

[b36] Wu G., Swaidan R., Li D. Y. & Li N. Enhanced methanol electro-oxidation activity of PtRu catalysts supported on heteroatom-doped carbon. Electrochim. Acta 53, 7622–7629 (2008).

[b37] Sevilla M., Valle-Vigón P. & Fuertes A. B. N-doped polypyrrole-based porous carbons for CO_2_ capture. Adv. Funct. Mater. 21, 2781–2787 (2011).

[b38] Li Z. L., Liu J. H., Xia C. G. & Li F. W. Nitrogen-functionalized ordered mesoporous carbons as multifunctional supports of ultrasmall Pd nanoparticles for hydrogenation of phenol. ACS Catal. 3, 2440–2448 (2013).

[b39] Kim S. J. *et al.* Highly active and CO_2_ tolerant Ir nanocatalysts for H_2_/CO_2_ separation in electrochemical hydrogen pumps. Appl. Catal. B 158–159, 348–354 (2014).

[b40] Xu D. *et al.* Iridium oxide nanoparticles and iridium/iridium oxide nanocomposites: photochemical fabrication and application in catalytic reduction of 4-nitrophenol. ACS Appl. Mater. Interfaces 7, 16738–16749 (2015). 26158693 10.1021/acsami.5b04504

[b41] Shen S. Y., Zhao T. S. & Xu J. B. Carbon-supported bimetallic PdIr catalysts for ethanol oxidation in alkaline media. Electrochim. Acta 55, 9179–9184 (2010).

[b42] Teterycz H., Klimkiewicz R. & Łaniecki M. Study on physico–chemical properties of tin dioxide based gas sensitive materials used in condensation reactions of n-butanol. Appl. Catal. A 274, 49–60 (2004).

[b43] Liang N., Zhang X. L., An H. L., Zhao X. Q. & Wang Y. J. Direct synthesis of 2-ethylhexanol via n-butanal aldol condensation–hydrogenation reaction integration over a Ni/Ce-Al_2_O_3_ bifunctional catalyst. Green Chem. 17, 2959–2972 (2015).

[b44] Xiong B. *et al.* The use of nitrogen-doped graphene supporting Pt nanoparticles as a catalyst for methanol electrocatalytic oxidation. Carbon 52, 181–192 (2013).

[b45] Wang Y., Yao J., Li H. R., Su D. S. & Antoniett M. Highly selective hydrogenation of phenol and derivatives over a Pd@carbon nitride catalyst in aqueous media. J. Am. Chem. Soc. 133, 2362–2365 (2011). 21294506 10.1021/ja109856y

[b46] Haque E., Jun J. W., Talapaneni S. N., Vinu A. & Jhung S. H. Superior adsorption capacity of mesoporous carbon nitride with basic CN framework for phenol. J. Mater. Chem. 20, 10801–10803 (2010).

